# 

**Published:** 1916-07

**Authors:** 


					﻿Vol. XXXII
JULY. 1916
No. 1
TEXAS
Medical Journal 1885
established july, 
Published monthly at austin,Texas, by
MRS. F. E, DANIEL Published monthl
VBDSD VB
VDADBSD  WRQ3   ETR R 
				

